# *N*′-Acetyl-5-amino-1-methyl-1*H*-pyrazole-4-carbohydrazonamide dihydrate

**DOI:** 10.1107/S1600536810015357

**Published:** 2010-04-30

**Authors:** Anna V. Dolzhenko, Anton V. Dolzhenko, Geok Kheng Tan, Lip Lin Koh, Giorgia Pastorin

**Affiliations:** a Department of Pharmacy, Faculty of Science, National University of Singapore, 18 Science Drive 4, Singapore 117543, Singapore; b Department of Chemistry, Faculty of Science, National University of Singapore, 3 Science Drive 3, Singapore 117543, Singapore

## Abstract

In the title compound, C_7_H_12_N_6_O·2H_2_O, the *Z* configuration of the hydrazone fragment is stabilized by an intra­molecular N—H⋯N hydrogen bond involving one of the amino groups. In the crystal structure, the hydrazonamide mol­ecules are connected *via* inter­molecular N—H⋯O=C hydrogen bonds, forming *C*(7) chains running along [010]. The chains form sheets parallel to the (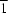
01). The chains are cross-linked by water mol­ecules to form a three-dimensional hydrogen-bonded network.

## Related literature

For bioactive pyrazoles, see: Elguero *et al.* (2002 ▶); Lamberth (2007 ▶). For the use of pyrazoles as synthons in heterocyclic chemistry, see: Schenone *et al.* (2007 ▶); Dolzhenko *et al.* (2008 ▶). For the use of pyrazoles in metal-organic chemistry, see: Mukherjee (2000 ▶); Halcrow (2009 ▶). For the crystal structures of related 5-amino-1*H*-pyrazole-4-carboxylic acid derivatives, see: Zia-ur-Rehman *et al.* (2008 ▶, 2009 ▶); Caruso *et al.* (2009 ▶). For the crystal structure of *N*′-acetyl-2-phenyl­ethane­hydra­zo­namide, see: Ianelli *et al.* (2001 ▶). For the graph-set analysis of hydrogen bonding, see: Bernstein *et al.* (1995 ▶).
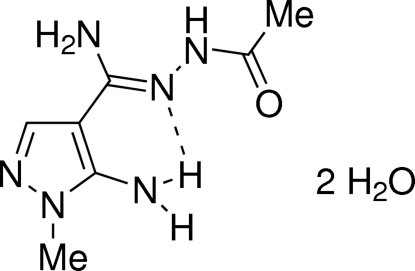


## Experimental

### 

#### Crystal data

C_7_H_12_N_6_O·2H_2_O
*M* *_r_* = 232.26
Triclinic, 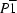

*a* = 7.5496 (9) Å
*b* = 7.6208 (9) Å
*c* = 11.2518 (13) Å
α = 102.645 (2)°
β = 101.440 (2)°
γ = 110.810 (2)°
*V* = 562.75 (11) Å^3^
*Z* = 2
Mo *K*α radiation
μ = 0.11 mm^−1^
*T* = 223 K
0.45 × 0.12 × 0.10 mm

#### Data collection

Bruker SMART APEX CCD diffractometer
Absorption correction: multi-scan (*SADABS*; Sheldrick, 2001 ▶) *T*_min_ = 0.953, *T*_max_ = 0.989
3963 measured reflections
2548 independent reflections
2174 reflections with *I* > 2σ(*I*)
*R*_int_ = 0.021

#### Refinement

*R*[*F* ^2^ > 2σ(*F* ^2^)] = 0.052
*wR*(*F* ^2^) = 0.141
*S* = 1.05
2548 reflections
183 parameters
H atoms treated by a mixture of independent and constrained refinement
Δρ_max_ = 0.31 e Å^−3^
Δρ_min_ = −0.26 e Å^−3^

### 

Data collection: *SMART* (Bruker, 2001 ▶); cell refinement: *SAINT* (Bruker, 2001 ▶); data reduction: *SAINT*; program(s) used to solve structure: *SHELXS97* (Sheldrick, 2008 ▶); program(s) used to refine structure: *SHELXL97* (Sheldrick, 2008 ▶); molecular graphics: *SHELXTL* (Sheldrick, 2008 ▶); software used to prepare material for publication: *SHELXTL*.

## Supplementary Material

Crystal structure: contains datablocks I, global. DOI: 10.1107/S1600536810015357/ci5086sup1.cif

Structure factors: contains datablocks I. DOI: 10.1107/S1600536810015357/ci5086Isup2.hkl

Additional supplementary materials:  crystallographic information; 3D view; checkCIF report

## Figures and Tables

**Table 1:** Hydrogen-bond geometry (Å, °)

*D*—H⋯*A*	*D*—H	H⋯*A*	*D*⋯*A*	*D*—H⋯*A*
O2*W*—H4*W*⋯N1^i^	0.87 (3)	2.04 (3)	2.884 (2)	162 (3)
O2*W*—H3*W*⋯O1	0.86 (3)	2.11 (3)	2.885 (2)	150 (3)
O1*W*—H2*W*⋯O2*W*^ii^	0.89 (3)	1.93 (3)	2.824 (2)	175 (3)
O1*W*—H1*W*⋯N5	0.81 (3)	2.24 (3)	2.982 (2)	153 (3)
N6—H6*N*⋯O1*W*^iii^	0.84 (2)	2.07 (2)	2.905 (2)	177 (2)
N4—H42⋯O1*W*^iii^	0.88 (2)	2.14 (3)	2.995 (2)	165 (2)
N4—H41⋯O1^iv^	0.81 (2)	2.08 (2)	2.874 (2)	169 (2)
N3—H32⋯N5	0.86 (2)	2.18 (2)	2.791 (2)	128 (2)
N3—H31⋯O2*W*^v^	0.83 (2)	2.27 (2)	3.082 (2)	163 (2)

Symmetry codes: (i) 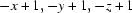; (ii) 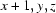; (iii) 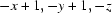; (iv) 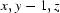; (v) 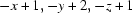.

**Fig. 1.**
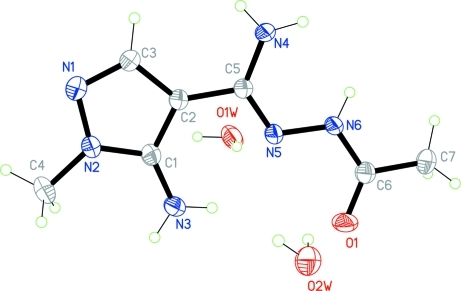
The molecular structure of N'-acetyl-5-amino-1-methyl-1H-pyrazole-4-carbohydrazonamide dihydrate showing the atomic numbering scheme. Displacement ellipsoids are drawn at the 50% probability level.

**Fig. 2.**
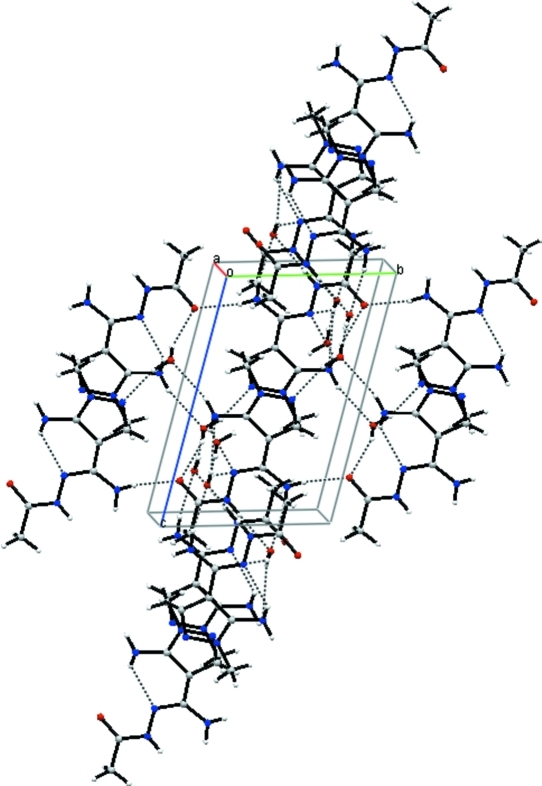
Crystal packing of the title compound, viewed along the a axis.

**Fig. 3.**
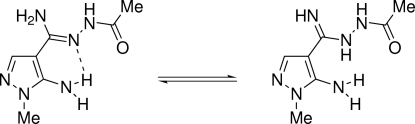
Hydrazonamide-imidohydrazide tautomerism in N'-acetyl-5-amino-1-methyl-1H-pyrazole-4-carbohydrazonamide

## supplementary crystallographic information

### Comment

Pyrazoles have been well recognized as valuable ligands in metal-organic chemistry (Mukherjee, 2000; Halcrow, 2009). Pyrazoles also possess useful agricultural (Lamberth, 2007) and pharmacological (Elguero *et al.*, 2002) properties and serve as synthons for other pyrazolo fused bioactive heterocycles (Schenone *et al.*, 2007; Dolzhenko *et al.*, 2008).

Herein, we report molecular and crystal structure of *N*'-acetyl-5-amino-1-methyl-1*H*-pyrazole-4-carbohydrazonamide (Figs. 1 and 2). The compound can exist in two tautomeric forms, namely hydrazonamide and imidohydrazide (Fig. 3). The hydrazonamide tautomer can also exhibit (*E*-*Z*) isomerism by inversion of configuration of the hydrazono C═N linkage. We found that the compound crystallizes as a *N*'-acetyl-5-amino-1-methyl-1*H*-pyrazole-4-carbohydrazonamide tautomer. Similarly to previously reported *N*'-acetyl-2-phenylethanehydrazonamide (Ianelli *et al.*, 2001), the hydrazonamide group of *N*'-acetyl-5-amino-1-methyl-1*H*-pyrazole-4-carbohydrazonamide adopts (*Z*)-configuration. This configuration is stabilized by the intramolecular N(3)H···N5═C5 hydrogen bonding between the amino group and the hydrazone N5 atom, generating an *S*(6) graph-set motif (Bernstein *et al.*, 1995). Similar NH···O═C interactions were reported for the structurally related derivatives of 5-amino-1*H*-pyrazole-4-carboxylic acid (Zia-ur-Rehman *et al.*, 2008; Zia-ur-Rehman *et al.*, 2009; Caruso *et al.*, 2009). Planarity of the molecule is affected by slight twisting of the acetyl group [C5—N5—N6—C6 torsion angle is 170.14 (16)°].

In the crystal, the hydrazonamide molecules are arranged to form sheets parallel to the (101) (Fig. 2). In the sheets, atom N4 of one molecule is involved in a intermolecular N—H···O═C interaction with the carbonyl atom O1 of adjacent molecule making *C*(7) chains along the [010] direction. The water molecules further stabilize packing by formation of the intermolecular hydrogen bond network (Fig. 2 and Table 1).

### Experimental

*N*'-Acetyl-5-amino-1-methyl-1*H*-pyrazole-4-carbohydrazonamide was prepared by treatment of ethyl *N*-(4-cyano-1-methyl-1*H*-pyrazol-5-yl)acetimidate with 3 eq. of hydrazine hydrate (40%) in ethanol. Detail procedure with proposed mechanism will be reported elsewhere. Single crystals suitable for the crystallographic analysis were grown by recrystallization from ethanol, m.p. 513 K.

### Refinement

All C-bound H atoms were positioned geometrically and included in the refinement in riding-motion approximation [0.95 Å for C_pyrazole_–H, and 0.98 Å for methyl groups; U_iso_(H) = 1.2U_eq_(C_pyrazole_) and U_iso_(H) = 1.5U_eq_(C_methyl_)] while the N- and O-bound H atoms were located in a difference map and refined freely.

### Crystal data

**Table:**

C_7_H_12_N_6_O·2H_2_O	*Z* = 2
*M**_r_* = 232.26	*F*(000) = 248
Triclinic, *P*1	*D*_x_ = 1.371 Mg m^−^^3^
Hall symbol: -P 1	Melting point: 513 K
*a* = 7.5496 (9) Å	Mo *K*α radiation, λ = 0.71073 Å
*b* = 7.6208 (9) Å	Cell parameters from 1515 reflections
*c* = 11.2518 (13) Å	θ = 3.0–27.5°
α = 102.645 (2)°	µ = 0.11 mm^−^^1^
β = 101.440 (2)°	*T* = 223 K
γ = 110.810 (2)°	Rod, colourless
*V* = 562.75 (11) Å^3^	0.45 × 0.12 × 0.10 mm

### Data collection

**Table:**

Bruker SMART APEX CCD diffractometer	2548 independent reflections
Radiation source: fine-focus sealed tube	2174 reflections with *I* > 2σ(*I*)
graphite	*R*_int_ = 0.021
φ and ω scans	θ_max_ = 27.5°, θ_min_ = 3.0°
Absorption correction: multi-scan (*SADABS*; Sheldrick, 2001)	*h* = −9→9
*T*_min_ = 0.953, *T*_max_ = 0.989	*k* = −9→9
3963 measured reflections	*l* = −14→13

### Refinement

**Table:**

Refinement on *F*^2^	Primary atom site location: structure-invariant direct methods
Least-squares matrix: full	Secondary atom site location: difference Fourier map
*R*[*F*^2^ > 2σ(*F*^2^)] = 0.052	Hydrogen site location: inferred from neighbouring sites
*wR*(*F*^2^) = 0.141	H atoms treated by a mixture of independent and constrained refinement
*S* = 1.05	*w* = 1/[σ^2^(*F*_o_^2^) + (0.0711*P*)^2^ + 0.1961*P*] where *P* = (*F*_o_^2^ + 2*F*_c_^2^)/3
2548 reflections	(Δ/σ)_max_ = 0.001
183 parameters	Δρ_max_ = 0.31 e Å^−^^3^
0 restraints	Δρ_min_ = −0.25 e Å^−^^3^

### Special details

**Table:**

Geometry. All esds (except the esd in the dihedral angle between two l.s. planes) are estimated using the full covariance matrix. The cell esds are taken into account individually in the estimation of esds in distances, angles and torsion angles; correlations between esds in cell parameters are only used when they are defined by crystal symmetry. An approximate (isotropic) treatment of cell esds is used for estimating esds involving l.s. planes.
Refinement. Refinement of *F*^2^ against ALL reflections. The weighted *R*-factor *wR* and goodness of fit *S* are based on *F*^2^, conventional *R*-factors *R* are based on *F*, with *F* set to zero for negative *F*^2^. The threshold expression of *F*^2^ > 2σ(*F*^2^) is used only for calculating *R*-factors(gt) *etc*. and is not relevant to the choice of reflections for refinement. *R*-factors based on *F*^2^ are statistically about twice as large as those based on *F*, and *R*- factors based on ALL data will be even larger.

### Fractional atomic coordinates and isotropic or equivalent isotropic displacement parameters (Å^2^)

**Table:**

	*x*	*y*	*z*	*U*_iso_*/*U*_eq_	
O1	0.3615 (2)	0.89463 (19)	0.14623 (12)	0.0421 (4)	
N1	0.7337 (2)	0.3950 (2)	0.48625 (14)	0.0306 (4)	
N2	0.7791 (2)	0.5933 (2)	0.51428 (13)	0.0264 (3)	
N3	0.7066 (3)	0.8221 (2)	0.42298 (16)	0.0302 (4)	
H31	0.748 (3)	0.907 (3)	0.495 (2)	0.036 (6)*	
H32	0.613 (3)	0.821 (3)	0.365 (2)	0.039 (6)*	
N4	0.3308 (2)	0.2471 (2)	0.11287 (15)	0.0297 (4)	
H41	0.350 (3)	0.158 (3)	0.132 (2)	0.032 (5)*	
H42	0.260 (3)	0.223 (3)	0.034 (2)	0.040 (6)*	
N5	0.4378 (2)	0.5945 (2)	0.18078 (13)	0.0294 (4)	
N6	0.3198 (2)	0.5816 (2)	0.06398 (13)	0.0267 (3)	
H6N	0.269 (3)	0.479 (3)	0.000 (2)	0.037 (6)*	
C1	0.6808 (2)	0.6344 (2)	0.41838 (15)	0.0239 (4)	
C2	0.5664 (2)	0.4541 (2)	0.32050 (15)	0.0236 (4)	
C3	0.6068 (3)	0.3143 (3)	0.37018 (16)	0.0272 (4)	
H3	0.5496	0.1780	0.3256	0.033*	
C4	0.9204 (3)	0.7328 (3)	0.63443 (17)	0.0359 (4)	
H4A	1.0140	0.8443	0.6188	0.054*	
H4C	0.9916	0.6687	0.6766	0.054*	
H4D	0.8504	0.7791	0.6886	0.054*	
C5	0.4374 (2)	0.4304 (2)	0.19753 (15)	0.0225 (3)	
C6	0.2965 (3)	0.7431 (3)	0.05365 (16)	0.0278 (4)	
C7	0.1873 (3)	0.7318 (3)	−0.07661 (17)	0.0354 (4)	
H7A	0.0729	0.7603	−0.0721	0.053*	
H7B	0.1431	0.6000	−0.1353	0.053*	
H7C	0.2751	0.8278	−0.1066	0.053*	
O1W	0.8443 (2)	0.7733 (2)	0.15574 (13)	0.0380 (4)	
H1W	0.752 (5)	0.757 (4)	0.186 (3)	0.069 (9)*	
H2W	0.949 (5)	0.780 (4)	0.213 (3)	0.065 (8)*	
O2W	0.1806 (2)	0.8169 (2)	0.34309 (14)	0.0393 (4)	
H3W	0.253 (4)	0.812 (4)	0.294 (3)	0.064 (9)*	
H4W	0.189 (4)	0.731 (4)	0.381 (3)	0.060 (8)*	

### Atomic displacement parameters (Å^2^)

**Table:**

	*U*^11^	*U*^22^	*U*^33^	*U*^12^	*U*^13^	*U*^23^
O1	0.0739 (10)	0.0235 (7)	0.0262 (7)	0.0259 (7)	0.0028 (7)	0.0046 (5)
N1	0.0382 (8)	0.0292 (8)	0.0260 (8)	0.0170 (7)	0.0052 (6)	0.0107 (6)
N2	0.0311 (7)	0.0255 (7)	0.0195 (7)	0.0118 (6)	0.0023 (6)	0.0060 (6)
N3	0.0413 (9)	0.0219 (7)	0.0218 (8)	0.0130 (7)	0.0021 (7)	0.0032 (6)
N4	0.0436 (9)	0.0173 (7)	0.0228 (8)	0.0136 (6)	−0.0018 (6)	0.0053 (6)
N5	0.0403 (8)	0.0219 (7)	0.0197 (7)	0.0141 (6)	−0.0034 (6)	0.0043 (6)
N6	0.0375 (8)	0.0195 (7)	0.0174 (7)	0.0128 (6)	−0.0022 (6)	0.0029 (6)
C1	0.0268 (8)	0.0265 (8)	0.0186 (7)	0.0119 (7)	0.0058 (6)	0.0071 (6)
C2	0.0286 (8)	0.0225 (8)	0.0199 (8)	0.0119 (6)	0.0046 (6)	0.0072 (6)
C3	0.0342 (9)	0.0228 (8)	0.0241 (8)	0.0138 (7)	0.0037 (7)	0.0076 (6)
C4	0.0374 (10)	0.0406 (11)	0.0206 (8)	0.0142 (8)	−0.0003 (7)	0.0043 (8)
C5	0.0274 (8)	0.0211 (8)	0.0188 (7)	0.0114 (6)	0.0045 (6)	0.0061 (6)
C6	0.0353 (9)	0.0264 (8)	0.0217 (8)	0.0143 (7)	0.0048 (7)	0.0085 (7)
C7	0.0447 (11)	0.0370 (10)	0.0273 (9)	0.0227 (9)	0.0026 (8)	0.0136 (8)
O1W	0.0406 (8)	0.0395 (8)	0.0236 (7)	0.0125 (6)	0.0021 (6)	0.0044 (6)
O2W	0.0518 (9)	0.0414 (8)	0.0299 (7)	0.0253 (7)	0.0082 (7)	0.0145 (6)

### Geometric parameters (Å, °)

**Table:**

O1—C6	1.236 (2)	C1—C2	1.401 (2)
N1—C3	1.317 (2)	C2—C3	1.402 (2)
N1—N2	1.372 (2)	C2—C5	1.459 (2)
N2—C1	1.344 (2)	C3—H3	0.94
N2—C4	1.445 (2)	C4—H4A	0.97
N3—C1	1.362 (2)	C4—H4C	0.97
N3—H31	0.83 (2)	C4—H4D	0.97
N3—H32	0.86 (2)	C6—C7	1.500 (2)
N4—C5	1.350 (2)	C7—H7A	0.97
N4—H41	0.81 (2)	C7—H7B	0.97
N4—H42	0.88 (2)	C7—H7C	0.97
N5—C5	1.303 (2)	O1W—H1W	0.81 (3)
N5—N6	1.3953 (19)	O1W—H2W	0.89 (3)
N6—C6	1.330 (2)	O2W—H3W	0.86 (3)
N6—H6N	0.84 (2)	O2W—H4W	0.87 (3)
			
C3—N1—N2	104.63 (14)	C2—C3—H3	123.7
C1—N2—N1	112.10 (14)	N2—C4—H4A	109.5
C1—N2—C4	127.04 (15)	N2—C4—H4C	109.5
N1—N2—C4	120.85 (14)	H4A—C4—H4C	109.5
C1—N3—H31	117.5 (15)	N2—C4—H4D	109.5
C1—N3—H32	110.8 (16)	H4A—C4—H4D	109.5
H31—N3—H32	119 (2)	H4C—C4—H4D	109.5
C5—N4—H41	116.7 (15)	N5—C5—N4	126.14 (15)
C5—N4—H42	123.9 (15)	N5—C5—C2	114.92 (14)
H41—N4—H42	118 (2)	N4—C5—C2	118.95 (15)
C5—N5—N6	117.52 (14)	O1—C6—N6	121.90 (15)
C6—N6—N5	117.50 (14)	O1—C6—C7	121.68 (16)
C6—N6—H6N	119.6 (15)	N6—C6—C7	116.42 (15)
N5—N6—H6N	122.8 (15)	C6—C7—H7A	109.5
N2—C1—N3	122.61 (15)	C6—C7—H7B	109.5
N2—C1—C2	106.59 (14)	H7A—C7—H7B	109.5
N3—C1—C2	130.72 (15)	C6—C7—H7C	109.5
C1—C2—C3	104.15 (14)	H7A—C7—H7C	109.5
C1—C2—C5	125.02 (15)	H7B—C7—H7C	109.5
C3—C2—C5	130.83 (15)	H1W—O1W—H2W	110 (3)
N1—C3—C2	112.53 (15)	H3W—O2W—H4W	103 (3)
N1—C3—H3	123.7		
			
C3—N1—N2—C1	0.66 (19)	N2—N1—C3—C2	−0.1 (2)
C3—N1—N2—C4	−178.36 (16)	C1—C2—C3—N1	−0.5 (2)
C5—N5—N6—C6	170.14 (16)	C5—C2—C3—N1	179.83 (17)
N1—N2—C1—N3	−177.99 (15)	N6—N5—C5—N4	−1.0 (3)
C4—N2—C1—N3	1.0 (3)	N6—N5—C5—C2	178.89 (14)
N1—N2—C1—C2	−0.95 (19)	C1—C2—C5—N5	1.9 (2)
C4—N2—C1—C2	178.00 (16)	C3—C2—C5—N5	−178.43 (17)
N2—C1—C2—C3	0.82 (18)	C1—C2—C5—N4	−178.18 (16)
N3—C1—C2—C3	177.53 (18)	C3—C2—C5—N4	1.5 (3)
N2—C1—C2—C5	−179.44 (15)	N5—N6—C6—O1	−6.2 (3)
N3—C1—C2—C5	−2.7 (3)	N5—N6—C6—C7	173.75 (16)

### Hydrogen-bond geometry (Å, °)

**Table:**

*D*—H···*A*	*D*—H	H···*A*	*D*···*A*	*D*—H···*A*
O2W—H4W···N1^i^	0.87 (3)	2.04 (3)	2.884 (2)	162 (3)
O2W—H3W···O1	0.86 (3)	2.11 (3)	2.885 (2)	150 (3)
O1W—H2W···O2W^ii^	0.89 (3)	1.93 (3)	2.824 (2)	175 (3)
O1W—H1W···N5	0.81 (3)	2.24 (3)	2.982 (2)	153 (3)
N6—H6N···O1W^iii^	0.84 (2)	2.07 (2)	2.905 (2)	177 (2)
N4—H42···O1W^iii^	0.88 (2)	2.14 (3)	2.995 (2)	165 (2)
N4—H41···O1^iv^	0.81 (2)	2.08 (2)	2.874 (2)	169 (2)
N3—H32···N5	0.86 (2)	2.18 (2)	2.791 (2)	128 (2)
N3—H31···O2W^v^	0.83 (2)	2.27 (2)	3.082 (2)	163 (2)

Symmetry codes: (i) −*x*+1, −*y*+1, −*z*+1; (ii) *x*+1, *y*, *z*; (iii) −*x*+1, −*y*+1, −*z*; (iv) *x*, *y*−1, *z*; (v) −*x*+1, −*y*+2, −*z*+1.
